# SARS-CoV-2 Vaccine Uptake among Patients with Chronic Liver Disease: A Cross-Sectional Analysis in Hebei Province, China

**DOI:** 10.3390/vaccines11081293

**Published:** 2023-07-28

**Authors:** Yongmei Liu, Wenfang Yuan, Haoting Zhan, Haiyan Kang, Xiaomeng Li, Yongliang Chen, Haolong Li, Xingli Sun, Linlin Cheng, Haojie Zheng, Wei Wang, Xinru Guo, Yongzhe Li, Erhei Dai

**Affiliations:** 1 Department of Clinical Laboratory, State Key Laboratory of Complex, Severe and Rare Diseases, Peking Union Medical College Hospital, Peking Union Medical College, Chinese Academy of Medical Sciences, Beijing 100730, China; yongmeiliude@163.com (Y.L.); haotingzhanpumch@126.com (H.Z.); lixiaomeng199107@sina.com (X.L.); jack3505@126.com (H.L.); liliacheng@126.com (L.C.); 2 Division of Liver Diseases, The Fifth Hospital of Shijiazhuang, Hebei Medical University, Shijiazhuang 050021, China; ywflzy@126.com (W.Y.); kanghaiyan1976@126.com (H.K.); 18132631589@163.com (Y.C.); 13363888159@163.com (X.S.); 13313019623@163.com (H.Z.); ww13216693@163.com (W.W.); gxr1998momoko@163.com (X.G.); 3 Department of Clinical Laboratory, Peking University People’s Hospital, Beijing 100035, China

**Keywords:** severe acute respiratory syndrome coronavirus 2 (SARS-CoV-2), coronavirus disease 2019 (COVID-19), vaccine, survey, chronic liver disease

## Abstract

Chronic liver disease (CLD) patients have higher mortality and hospitalization rates after infection with severe acute respiratory syndrome coronavirus 2 (SARS-CoV-2). This study aimed to explore SARS-CoV-2 vaccine perceptions, side effects, factors associated with nonvaccination and attitudes toward fourth-dose vaccine among CLD patients. The differences between vaccinated and unvaccinated groups among 1491 CLD patients and the risk factors associated with nonvaccination status were analyzed. In total, 1239 CLD patients were immunized against SARS-CoV-2. CLD patients have a high level of trust in the government and clinicians and were likely to follow their recommendations for vaccination. Reasons reported for nonvaccination were mainly concerns about the vaccines affecting their ongoing treatments and the fear of adverse events. However, only 4.84% of patients reported mild side effects. Risk factors influencing nonvaccination included being older in age, having cirrhosis, receiving treatments, having no knowledge of SARS-CoV-2 vaccine considerations and not receiving doctors’ positive advice on vaccination. Furthermore, 20.6% of completely vaccinated participants refused the fourth dose because they were concerned about side effects and believed that the complete vaccine was sufficiently protective. Our study proved that SARS-CoV-2 vaccines were safe for CLD patients. Our findings suggest that governments and health workers should provide more SARS-CoV-2 vaccination information and customize strategies to improve vaccination coverage and enhance vaccine protection among the CLD population.

## 1. Background

Severe acute respiratory syndrome coronavirus 2 (SARS-CoV-2) is a highly contagious virus that causes damage to the respiratory tract and extrarespiratory organs (such as the heart, muscles and nervous and digestive systems), and systemic symptoms (e.g., chill, fatigue, malaise and fever). SARS-CoV-2 resulted in a worldwide pandemic in 2020 in the human population. It has previously been reported that SARS-CoV-2 could cause interferon inhibition and a cytokine storm, thereby leading to an overaggressive immune response. The increased binding affinity of the SARS-CoV-2 variants (including Alpha (B.1.1.7), Beta (B.1.351), Gamma (P.1), Delta (B.1.617.2) and Omicron (B.1.1.529)) to the host receptor or their ability to evade the natural or vaccine-induced immunity has increased the virulence and transmission of the virus, resulting in several waves of infections worldwide [1]. It is well-known that herd or community immunity can be established through vaccination to protect the vulnerable [2]. By 20 January 2023, 3.488 billion doses of SARS-CoV-2 vaccines had been reported in the Chinese mainland (https://www.chinacdc.cn/jkzt/crb/zl/szkb_11803/jszl_13141/202301/t20230125_263519.html, accessed on 27 July 2023). However, there were no data on the vaccination rate against SARS-CoV-2 in chronic liver disease (CLD) patients.

CLD patients have a higher risk of death and hospitalization after SARS-CoV-2 infection [3]. A previous study reported that SARS-CoV-2 infection was related to a 3.5-fold increase in mortality in cirrhosis patients [4]. Alcohol-related liver disease (ALD) was found to be the independent risk factor for death (OR = 1.79) in coronavirus disease 2019 (COVID-19) patients [5]. Moreover, patients infected with SARS-CoV-2 plus the hepatitis C virus were more likely to be hospitalized [6]. According to the American Association for the Study of Liver Diseases and the European Association for the Study of the Liver, CLD patients are included in the priority group to receive vaccines against SARS-CoV-2 [7,8]. One study found that the positive rate of serum-neutralizing antibodies was above 75% in CLD patients after two doses of inactive vaccine [9]. In addition, it has been reported that vaccination could reduce mortality in cirrhosis patients despite breakthrough infections [10]. However, Cao et al. [11] found that only 37.12% of cirrhosis patients received SARS-CoV-2 vaccines in Shanghai Ruijin Hospital. A USA-based study revealed that younger age, female gender, lower household income and self-report of not taking the annual influenza vaccine were independently associated with high vaccine hesitancy in patients with inflammatory bowel disease and cirrhosis and in liver transplant recipients [12]. Furthermore, another study demonstrated that the main factors hindering SARS-CoV-2 vaccination are concerns about the safety and side effects of the vaccines [13].

Currently, there are no data on the prevalence of vaccination against SARS-CoV-2 among CLD patients, and the safety of SARS-CoV-2 vaccines in CLD patients is largely unknown. This study explored SARS-CoV-2 vaccination rates, vaccine side effects, perceptions and concerns about vaccines, factors associated with unvaccinated status and attitudes to fourth-dose vaccines in a large CLD cohort in China.

## 2. Methods

### 2.1. Questionnaire and Participants

This study was performed in the Fifth Hospital of Shijiazhuang (Hebei Province, China) and involved CLD patients aged 18 years or over with either clinical or pathological diagnoses. A survey containing 51 items was designed and used to assess the CLD patients based on four domains: ① demographic and sociological information; ② cognition and attitude towards the SARS-CoV-2 vaccine; ③ SARS-CoV-2 vaccination status and vaccine safety assessment; and ④ the attitude towards the fourth dose of the SARS-CoV-2 vaccine. The questionnaire was available to all patients who visited the outpatient clinic or were admitted to the inpatient department; however, only CLD patients diagnosed by clinicians and those who volunteered to participate in this study filled out the questionnaire. A total of 1604 questionnaires were collected (https://www.wjx.cn/, accessed on 27 July 2023) from 7 July 2022 to 29 August 2022, among which 113 were excluded because patients were under 18 years old or the questionnaires were incomplete or filled out in duplicate. Finally, 1491 questionnaires were analyzed with a recovery rate of 92.96%. This study was approved by the Ethics Committee of the Fifth Hospital of Shijiazhuang (2022-017-1).

### 2.2. SARS-CoV-2 Vaccination Program

According to the Technical Guidelines for Vaccination of SARS-CoV-2 (First Edition) issued by the Chinese Health Commission, the SARS-CoV-2 vaccines approved in Mainland China include two doses of inactivated vaccine (Sinopharm/BBIBP-CorV, CoronaVac/Sinovac and Sinopharm/WIBP), one dose of viral-vector-based vaccine (Convidecia/CanSinoBio) and three doses of recombinant subunit vaccine (Zhifei Longcom, Hefei, China). The recommended interval between two doses of inactivated vaccine is at least three weeks, and the second dose should be administered as soon as possible, within eight weeks. On the other hand, the recommended interval between two adjacent doses of recombinant subunit vaccine is at least four weeks; the second dose should be completed within eight weeks of the first dose, and the third dose should be completed within six months of the first dose [14]. Complete vaccination means that a person has received one dose of the viral-vector-based vaccine, two doses of the inactivated vaccines or three doses of the recombinant subunit vaccines. Partial vaccination is when a person has received one dose of the inactivated vaccine or fewer than three doses of the recombinant subunit vaccines. A booster vaccination is defined as an additional vaccination with the same type of vaccine for people who have completed two doses of inactivated or one dose of viral-vector-based vaccine within six months (http://health.people.com.cn/n1/2021/1116/c14739-32283576.html, accessed on 27 July 2023). For sequential vaccination, people who have completed two doses of inactivated vaccine are revaccinated within six months with one dose of viral-vector-based vaccine or recombinant subunit vaccine (http://www.gov.cn/xinwen/2022-02/19/content_5674674.htm, accessed on 27 July 2023).

### 2.3. Statistical Analyses

Statistical analyses were performed using IBM SPSS Statistics (version 26.0) and GraphPad Prism (version 8.0) software. The Shapiro–Wilk test was applied to determine the normality of quantitative data. Median and interquartile ranges (IQR) presented non-normally distributed continuous variables, whereas counts and percentages indicated categorical variables. The Wilcoxon rank-sum test was used to analyze group differences for non-normally distributed values, whereas the Chi-squared test was employed to compare the differences of categorical variables between groups with and without SARS-CoV-2 vaccination.

With regard to univariable analyses, binary logistic regression models were used to calculate odds ratios (OR) and 95% confidence intervals (CI) to explore factors associated with nonvaccination against SARS-CoV-2. The following factors were evaluated in the univariable analyses: age (≥60 vs. <60), types of CLD (viral hepatitis, cirrhosis, autoimmune liver disease, ALD and nonalcoholic fatty liver disease (NAFLD)), liver disease course (<1 year, 1–5 years, 5–10 years and >10 years), treatments (antiviral drugs, immunosuppressants and Chinese patent medicine), comorbidities (cardiovascular and cerebrovascular diseases, respiratory diseases, diabetes and chronic kidney disease), allergy history (yes vs. no), education (having a university degree vs. not), SARS-CoV-2 vaccine considerations (know vs. not knowing), belief that SARS-CoV-2 vaccination affects treatment (agree vs. disagree and neutral) and receiving doctors’ positive advice on vaccination (vs. not). Moreover, statistically significant variables (*p* < 0.05) associated with nonvaccination status in the univariable analyses were further subjected to multivariable logistic regression analyses. Notably, the variance inflation factor was less than 3 and tolerance was greater than 0.3, indicating no multicollinearity. *p* < 0.05 was considered statistically significant.

## 3. Results

### 3.1. Participant Characteristics

Among the 1491 CLD patients included in this study, 83.10% were immunized against SARS-CoV-2, and 65.25% were men (Table 1). The age of participants ranged between 18 and 97 years (median = 46.00, IQR = 20.00). With regard to the etiology of liver disease, 1169 (78.4%) patients had viral hepatitis, 321 (21.5%) had cirrhosis, 36 (2.4%) had autoimmune liver diseases, 28 (1.9%) had NAFLD and 18 (1.2%) had ALD. In addition, 1.2% (18/1491) of CLD patients had breakthrough infections with SARS-CoV-2, and 1092 (73.2%) were worried about contracting SARS-CoV-2.

Demographic and clinical characteristics indicated that the group without SARS-CoV-2 vaccination was older (median = 56.00, IQR = 17.50, *p* < 0.001), with longer liver disease duration (*p* < 0.001), a higher proportion of cirrhosis (52.0% vs. 15.3%, *p* < 0.001), a higher number of treatments (94.8% vs. 80.4%, *p* < 0.001), allergy history (18.7% vs. 10.3%, *p* < 0.001), lower educational attainment (*p* < 0.001) and lower monthly family income (*p* < 0.001) compared to the vaccinated group. However, the two groups had no significant differences in sex, comorbidities, living area and marital status. Regarding the vaccine belief, there was a higher percentage of patients in the vaccinated group who thought that the SARS-CoV-2 vaccine was effective, safe and essential for the health of people around them compared to the unvaccinated group (*p* = 0.025, *p* < 0.001 and *p* = 0.004, respectively). In addition, 40.0% of the vaccinated patients disagreed that SARS-CoV-2 vaccination had adverse effects on liver disease and treatment, whereas only 19.8% of the unvaccinated group disagreed (*p* < 0.001).

### 3.2. Issues that CLD Patients Were Concerned about before SARS-CoV-2 Vaccination

**Figure 1:**
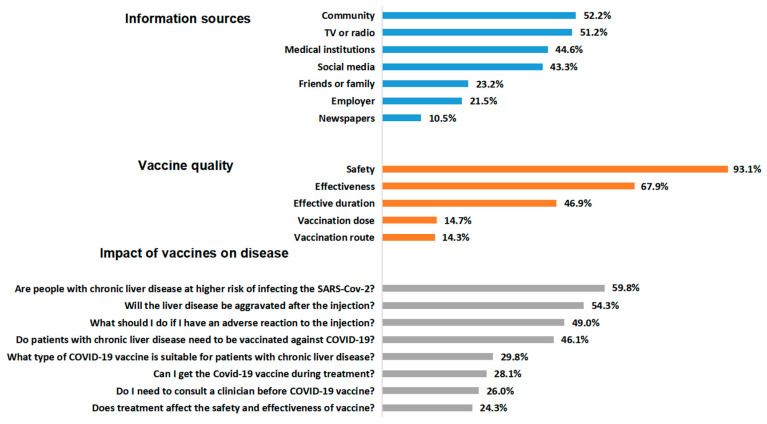
Information sources, vaccine quality and impact of vaccines on chronic liver disease (CLD). Abbreviations: COVID-19: coronavirus disease 2019; SARS-CoV-2: severe acute respiratory syndrome coronavirus 2.

Results showed that the participants received SARS-CoV-2 vaccine or vaccination information mainly from publicity committees (778, 52.2%), television or radio (763, 51.2%), medical institutions (665, 44.6%), social media (644, 43.3%), friends or family (346, 23.2%), employers (3211, 21.5%) and newspapers (157, 10.5%). The top three vaccine quality issues that participants were concerned about were safety (1388, 93.1%), effectiveness (1013, 67.9%) and effective duration of the SARS-CoV-2 vaccine (700, 46.9%). In terms of the impacts of vaccination on CLD, the top four concerns were as follows: “*Are CLD patients at higher risk of contracting SARS-CoV-2?*” (892, 59.8%), “*Will the disease be aggravated after the SARS-CoV-2 vaccination?*” (809, 54.3%), “*What should I do if I have an adverse reaction to the vaccine?*” (731, 49.0%) and “*Do CLD patients require vaccination against SARS-CoV-2?*” (688, 46.1%). These results are illustrated in Figure 1.

### 3.3. SARS-CoV-2 Vaccination Status

**Figure 2:**
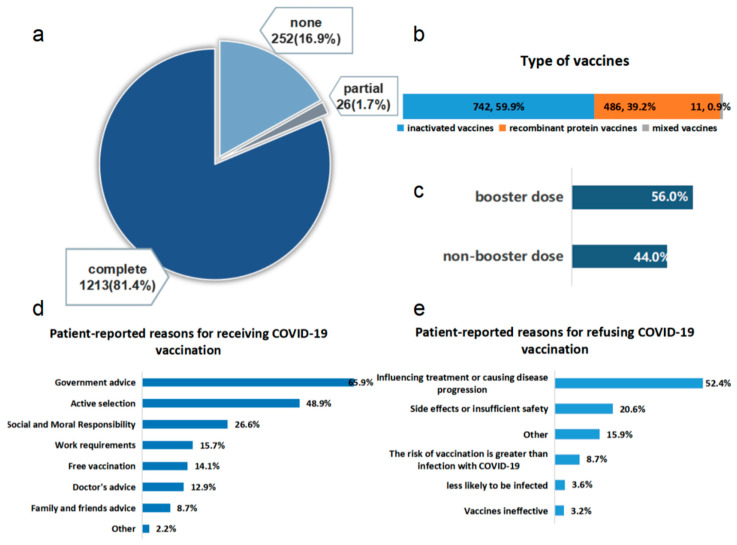
SARS-CoV-2 vaccination status and reasons for vaccinating or not vaccinating. (**a**) The SARS-CoV-2 vaccination rate among chronic liver disease (CLD) patients. (**b**) The types of vaccines administered to the vaccinated CLD patients. (**c**) The booster vaccination rate among the full-course vaccinated CLD patients. (**d**,**e**). Patients’ reported reasons for receiving (**d**) and refusing (**e**) COVID-19 vaccination.

As of 23 August 2022, 252 (16.9%) participants were unvaccinated against SARS-CoV-2. Results revealed that 26 (1.7%) participants received partial vaccination (one dose of inactivated vaccine or two doses of recombinant protein vaccine) of the SARS-CoV-2 vaccine, and 1213 (81.4%) participants received complete vaccination (two doses of inactivated vaccine and three doses of recombinant protein vaccine) (Figure 2a). Among the 1239 participants who received at least one dose of SARS-CoV-2 vaccine, 742 (59.9%) patients received inactivated vaccines, 486 (39.2%) received recombinant protein vaccines and 11 (0.9%) received sequential booster immunizations (first two doses were inactivated vaccines, but the third dose was either the recombinant protein vaccine or the adenovirus vaccine) (Figure 2b). Moreover, 679 (56.0%) fully vaccinated patients received the booster dose (Figure 2c).

The 1239 vaccinated participants attributed their willingness to be vaccinated mainly to government advice (816, 65.9%), active selection to prevent SARS-CoV-2 infection (606, 48.9%) and social and moral responsibility (330, 26.6%). In addition, 195 (15.7%), 175 (14.1%), 160 (12.9%) and 108 (8.7%) participants received SARS-CoV-2 vaccines due to work requirements, free vaccination, doctor’s advice and family and friend advice, respectively (Figure 2d). Among the 252 unvaccinated participants, 132 (52.4%) worried about the effects of the vaccines on their ongoing treatments or the possibility of the vaccines aggravating their disease progression, 52 (20.6%) were afraid of vaccine side effects or insufficient safety, 22 (8.7%) believed that the risk of vaccination was greater than infection with SARS-CoV-2, 9 (3.6%) believed that they were less likely to be infected with SARS-CoV-2 and 8 (3.2%) thought that the vaccines were ineffective. In addition, 40 (15.9%) participants were unvaccinated due to pregnancy, other chronic diseases, suspension of vaccinations suggested by doctor, allergic reactions to vaccine components, etc. (Figure 2e).

**Figure 3:**
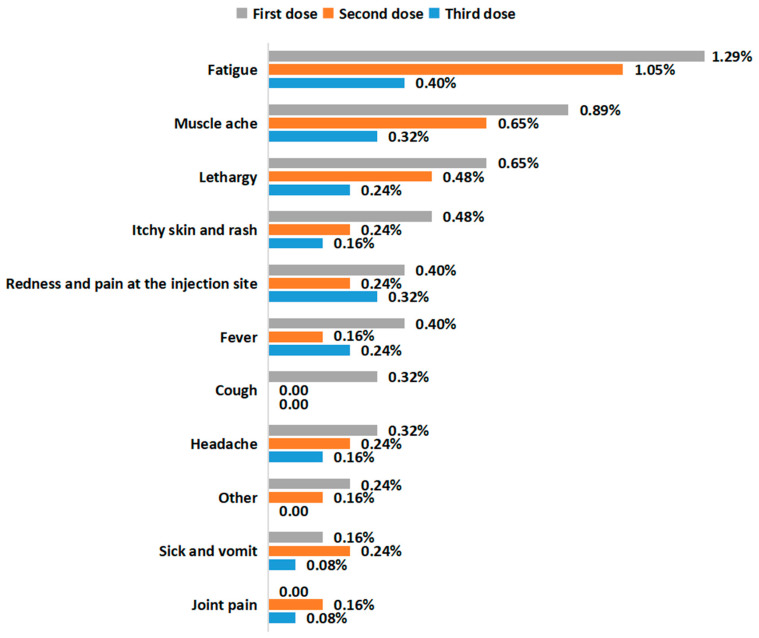
Adverse events after SARS-CoV-2 vaccination.

Safety analysis was performed on the 1239 participants who received at least one dose of the SARS-CoV-2 vaccine. Adverse events were only reported in 60 (4.84%) CLD patients after SARS-CoV-2 vaccination. Figure 3 shows the side effects associated with each vaccine dose. Results demonstrated that 33 (2.66%), 22 (1.78%) and 13 (1.05%) CLD patients reported side effects after the first dose, second dose and third dose, respectively. The most common adverse events after vaccination were fatigue, muscle ache and lethargy. Generally, the rate of side effects was lower after the third dose than after the first dose (*p* = 0.007). However, no significant difference was observed concerning the rate of side effects between the first and second doses and between the second and third doses (*p* = 0.155 and *p* = 0.174, respectively).

### 3.4. Risk Factors Associated with Unvaccinated Status

Univariable factors associated with unvaccinated status were older age (>60 years old), cirrhosis, autoimmune liver disease, long liver disease course (>1 year), receiving treatments (such as immunosuppressant and Chinese patent medicine), having comorbidities (cardiovascular and cerebrovascular diseases, respiratory diseases, diabetes and chronic kidney disease) and allergy history. Other factors included not having a university degree, not being knowledgeable about SARS-CoV-2 vaccination considerations, thinking SARS-CoV-2 vaccination affects treatment and not receiving doctors’ positive advice on vaccination (Table 2).

According to the multivariate logistic regression analysis, the statistically significant risk factors of unvaccinated status included older age (OR = 2.731, 95% CI: 1.811–4.12, *p* < 0.001); having cirrhosis (OR = 2.922, 95% CI: 1.972–4.331, *p* < 0.001); long liver disease course (compared to <1 year, 1–5 years: OR = 3.37, 95% CI: 1.782–6.374, *p* < 0.001; 5–10 years: OR = 3.326, 95% CI: 1.666–6.286, *p* = 0.001; >10 years: OR = 2.792, 95% CI: 1.495–5.216, *p* = 0.001); receiving treatments (OR = 2.471, 95% CI: 1.318–4.632, *p* = 0.005), especially of immunosuppressants (OR = 3.764, 95% CI: 1.083–13.08, *p* = 0.037); having comorbidities (OR = 2.004, 95% CI: 1.058–3.798, *p* = 0.033); and allergy history (OR = 1.732, 95% CI: 1.107–2.71, *p* = 0.016). Additional factors were no university degree (OR = 1.959, 95% CI: 1.187–3.231, *p* = 0.008), no knowledge about SARS-CoV-2 vaccine considerations (OR = 3.145, 95% CI: 2.253–4.391, *p* = 0.001), thinking SARS-CoV-2 vaccination affects treatment (OR = 1.89, 95% CI: 1.284–2.783, *p* < 0.001), and not receiving doctors’ advice on vaccination (OR = 3.145, 95% CI: 1.253–4.391, *p* < 0.001) (Table 2).

### 3.5. Attitudes to the Fourth Dose of the SARS-CoV-2 Vaccine

**Figure 4:**
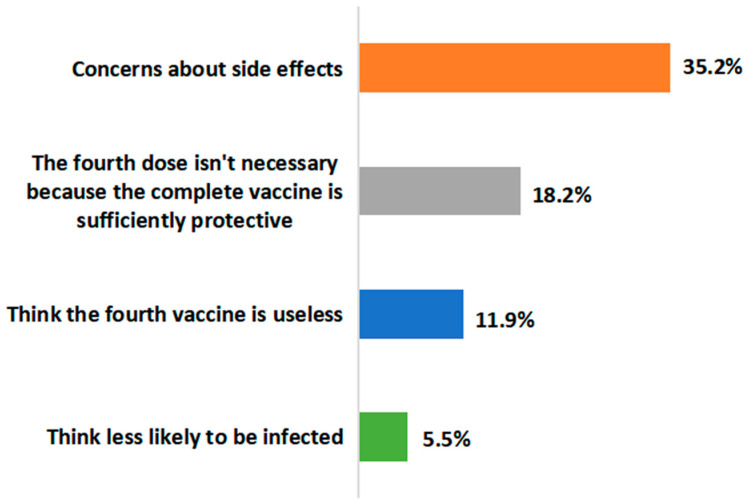
The reasons for refusing the fourth COVID-19 vaccine dose.

Among the 1144 CLD patients who received three vaccine doses, 908 (79.4%) were willing to accept the fourth vaccine dose, whereas 236 (20.6%) refused. There were no statistical differences in the acceptability of the fourth dose of the SARS-CoV-2 vaccine among participants receiving inactivated, recombinant protein and mixed vaccines (Table 3). Figure 4 illustrates factors associated with the unwillingness to accept the fourth dose of the SARS-CoV-2 vaccine. The most common reason was the concerns about side effects (35.2%), followed by the sense of sufficient protective effect of the already administered doses (18.2%). Furthermore, 11.9% of participants believed that the fourth dose was useless and 5.5% thought they were less likely to be infected with SARS-CoV-2.

## 4. Discussion

The SARS-CoV-2 vaccine has been demonstrated to be a safe and efficient tool to prevent severe SARS-CoV-2 infections, hospitalization and death [15]. In this study, we found that 81.4% of CLD patients from northern provinces of China received a complete dose of SARS-CoV-2 vaccines, and most patients held positive views and attitudes toward SARS-CoV-2 vaccination. However, the SARS-CoV-2 vaccination rate among CLD patients was lower than the overall 89.7% in China (http://sn.people.com.cn/n2/2022/0724/c186331-40051320.html, accessed on 27 July 2023). Therefore, we further explored the reasons and factors associated with nonvaccinating, with the overarching goal of developing strategies to improve the vaccination rate among CLD patients.

Efficacy and safety are the drivers of vaccination. This study found that concerns about the vaccines affecting ongoing treatments or aggravating disease progress and the fear of adverse events were the main reasons why CLD patients refused the vaccines. However, only a few (60/1239, 4.84%) adverse events were observed, mainly fatigue, lethargy, muscle ache and redness and pain at the injection site, which was consistent with other CLD cohorts [9,16,17,18]. Additionally, the types of adverse events occurring in CLD patients (such as fatigue, headache, pain, redness and swelling at the injection site) were analogous to those in healthy individuals [19]. Comparing the adverse events between CLD and non-CLD communities was difficult since the incidence rate varied widely in the general population among different studies, ranging from 0.01186% to 80.3%. The variation was due to the differences in the population size, vaccine brand, survey time, etc. (http://health.people.com.cn/n1/2021/0528/c14739-32116490.html, accessed on 27 July 2023) [20,21,22,23,24,25,26,27]. Moreover, considering the differences in physical function, the impact of the vaccines on liver function needed to be further analyzed in CLD patients to reflect vaccine safety. It was reported that among 103 hepatitis B patients with abnormally high function liver indexes after vaccination, 58 had normal liver function within the 6-month period before vaccination. However, 42.9% of the patients regained their normal liver function without additional treatment, and 57.1% only had mild liver abnormalities within the 6-month period after vaccination [28]. Aspartate aminotransferase, alanine aminotransferase (ALT) and total bilirubin (TBil) levels remained stable in hepatitis B patients and healthy volunteers after the first 3 months of vaccination [17]. Mild to moderate elevations of TBil and ALT levels without any clinical significance were observed in 8% of healthy recipients [29]. These cases of liver function abnormalities after vaccination against SARS-CoV-2 could be the result of a combination of factors and were self-limiting, which probably indicating that the adverse events were directly related to vaccines but not to the vaccinated population. The above results demonstrated that SARS-CoV-2 vaccines had a favorable safety profile in CLD patients.

Although some CLD patients (90% cirrhosis) and liver transplant recipients contracted SARS-CoV-2 infection after vaccination, unvaccinated individuals had higher rates of symptomatic infection, hospitalization, ICU admission, invasive ventilation and death than vaccinated CLD patients. A previous study reported that vaccination against SARS-CoV-2 could cause favorable outcomes for CLD patients [30]. Furthermore, patients with cirrhosis had lower vaccination rates (190/329, 58.11%) than patients with other CLDs, even though vaccination against SARS-CoV-2 in the cirrhosis population was shown to be safe [11]. Given that 63% of the vaccinated patients got vaccinated because of government advice, it is safe to speculate that most CLD patients have high levels of trust in the government. Therefore, spreading and explaining vaccine-associated knowledge through committees, television or radio, medical institutions and social media might alleviate patient doubts and concerns about SARS-CoV-2 vaccination.

Identifying risk factors associated with unvaccinated status is critical to customizing appropriate strategies and providing timely interventions. This study found that the cognition of CLD patients to SARS-CoV-2 vaccines and positive advice from medical workers influenced vaccination. The risk factors associated with unvaccinated status were the lack of knowledge about SARS-CoV-2 vaccination considerations, the belief that SARS-CoV-2 vaccination could negatively influence the treatment effect and the lack of receiving doctors’ positive advice on vaccination. It is worth noting that the American Association for the study of liver diseases expert panel released a consensus statement that suggested patients with CLD are among the priority population for SARS-CoV-2 vaccination, and healthcare providers should actively inform patients of vaccination-related information [7]. Herein, it was evident that patients who received positive advice from doctors on vaccination were more likely to accept the vaccines (90.11%, 938/1041) than those who did not receive doctors’ advice (66.89%, 301/450) (*p* < 0.001). Notably, a previous study proved that healthcare workers were more willing to be vaccinated than nonhealthcare workers [31], which implies the importance of clinicians’ advice and popularizing knowledge about SARS-CoV-2 vaccination. Moreover, we found that CLD patients over 60 years old, with cirrhosis, a liver disease course of more than one year, receiving treatments or having comorbidities or allergy history and having no university degree were more unwilling to accept the SARS-CoV-2 vaccine. This was consistent with previous studies that reported lower vaccine acceptance among the elderly and less educated population [32,33,34]. In addition, like the CLD patients (except for concerns about CLD aggravation), major reasons that hindered vaccination willingness among the unvaccinated general population were the fear of adverse events, mistrust of vaccines’ efficacy and safety, negative information about vaccinations or lack of knowledge on vaccination [35,36,37,38,39]. Collectively, these results suggest that governments and healthcare workers should make more flexible and comprehensive efforts to further advocate the necessity of vaccination.

The emergence of SARS-CoV-2 variants and decreased immunity necessitates enhancing the SARS-CoV-2 vaccine dose, but there are limited data on willingness to receive an additional vaccine. In this study, 20.63% of 1144 completely vaccinated participants were unwilling to accept the fourth dose since they believed the doses they had received were sufficiently protective. These results were similar to a previous report which indicated that 18.9% of the general population were hesitant about the vaccination due to their confidence in the current epidemic control, the effectiveness of previous vaccinations and the uncertainty about the need for extra protection [40]. However, a recent study reported that neutralizing-antibody titers in CLD patients decreased with prolonged vaccination, and the antibody-positive rates were 72.6%, 45.3% and 43.9% after the first, second and third months, respectively [17]. Davidov et al. [24] found that two doses of BNT162b2 mRNA vaccine could not produce sufficient protection among liver transplant recipients, and a third dose of the mRNA vaccine significantly improved the levels of neutralizing antibody, anti-RBD antibody and activated T cells. In addition, studies have revealed that antibodies produced after vaccination were low in CLD patients who received immunosuppressive therapy [41] and patients with cirrhosis who were male or had HBeAg and HBV DNA positive reaction or a high Child–Pugh score [18]. Therefore, to reduce the risk of infection and the occurrence of severe disease or death in CLD patients, it is essential to carry out immune monitoring work after the SARS-CoV-2 vaccination, identify patients with poor immune response and administer another dose of vaccine.

Notably, the number of vaccinated patients identified in our study (n = 1239) was much higher than that of unvaccinated patients (n = 252), indicating the SARS-CoV-2 vaccination rates among CLD patients (83.10%, 1239/1491) in the real world. This difference might be explained by several factors. First, SARS-CoV-2 vaccinations are provided for free to the public in China, which has been proven to increase the vaccination willingness rate [13]. Second, by the time this survey was conducted, the Chinese government had published the first edition of the SARS-CoV-2 vaccination technical guideline [42], and the European, American and Chinese experts successively published a consensus on the use of vaccines to prevent COVID-19 in patients with liver disease, which promoted and popularized SARS-CoV-2 vaccines. Third, as shown in our research, the Chinese people strongly trust their central government and are willing to get vaccinated to reduce the risk of infection. This group difference was also seen in the Chinese adult population [43] and individuals with cancer, autoimmune diseases or other serious comorbid conditions [44].

However, the present study had some limitations. First, although we tried to avoid inaccurately self-reported information by validating the provided data using the hospital information system and asking participants to submit vaccine records, there may be some recall bias in the cross-sectional survey design. There might have also been data exclusion, especially for some persons who could not complete the online questionnaire due to the lack of necessary gadgets such as smartphones. Second, there were few patients with autoimmune liver diseases and the percentage of those with ALD and NAFLD was small; therefore, no data were collected on the time and severity of side effects in these patients. Therefore, there is a need for larger multicenter cohort studies to validate our results. Third, despite the large sample size, this was a single-center study from the northern provinces of China during a period when COVID-19 was under control. Therefore, it is not clear whether our results could be extrapolated to other regions.

## 5. Conclusions

This study revealed that although SARS-CoV-2 vaccines are generally safe, the current acceptance rate of vaccination among CLD patients in China is relatively low. Therefore, governments, policymakers, healthcare workers and hepatology societies should dedicate more effort to addressing the vaccination gap in this population, especially patients at an older age; with cirrhosis, a long disease course, treatments, comorbidities, allergy history and no university degree; and who are not knowledgeable about SARS-CoV-2 vaccination. Furthermore, multicenter and multiregion studies should be conducted to develop strategies for increasing SARS-CoV-2 vaccination coverage among CLD patients.

## Figures and Tables

**Table 1:** Patient characteristics and vaccine beliefs stratified by COVID-19 vaccination condition.

	All Patients (n = 1491)	With COVID-19 Vaccination (n = 1239)	Without COVID-19 Vaccination (n = 252)	*p*-Value
**Age (years),** Median (IQR)	46.00 (20.00)	43.00 (19.00)	56.00 (17.50)	<0.001
**Male**, n (%)	973 (65.3%)	817 (65.9%)	156 (61.9%)	0.220
**Etiology of liver disease**, n (%)				<0.001
Hepatitis B/C	1169 (78.4%)	1006 (81.2%)	163 (64.7%)	
Autoimmune liver diseases	36 (2.4%)	23 (1.9%)	13 (5.2%)	
ALD	18 (1.2%)	14 (1.1%)	4 (1.6%)	
NAFLD	28 (1.9%)	26 (2.1%)	2 (0.8%)	
Hepatitis cirrhosis	152 (10.2%)	92 (7.4%)	60 (23.8%)	
Autoimmune cirrhosis	10 (0.7%)	2 (0.2%)	8 (3.2%)	
Alcoholic cirrhosis	9 (0.6%)	5 (0.4%)	4 (1.6%)	
Fatty cirrhosis	6 (0.4%)	5 (0.4%)	1 (0.4%)	
Other cirrhosis	153 (10.3%)	93 (7.5%)	60 (23.8%)	
Other liver diseases	33 (2.2%)	20 (1.6%)	13 (5.2%)	
**Liver disease course (years)**, n (%)				<0.001
<1 year	230 (15.4%)	214 (17.3%)	16 (6.4%)	
1–5 years	376 (25.2%)	297 (24.0%)	79 (31.4%)	
5–10 years	293 (19.7%)	236 (19.1%)	57 (22.6%)	
>10 years	592 (39.7%)	492 (39.7%)	100 (39.7%)	
**Treatments**, n (%)	1235 (82.8%)	996 (80.4%)	239 (94.8%)	<0.001
Antiviral drugs	1010 (67.7%)	833 (67.2%)	177 (70.2%)	
Immunosuppressants	14 (0.9%)	7 (0.6%)	7 (2.8%)	
Chinese patent medicines	237 (15.9%)	163 (13.2%)	74 (29.4%)	
Other treatments	81 (5.4%)	52 (4.2%)	29 (11.5%)	
**Comorbidities**, n (%)	247 (16.6%)	166 (13.4%)	81 (32.1%)	0.511
Cardiovascular and cerebrovascular diseases	128 (8.6%)	89 (7.2%)	39 (15.5%)	
Respiratory diseases	15 (1.0%)	9 (0.7%)	6 (2.4%)	
Diabetes	87 (5.8%)	51 (4.1%)	36 (14.3%)	
Chronic kidney disease	19 (1.3%)	12 (1.0%)	7 (2.8%)	
**Allergy history**, n (%)				<0.001
Yes	174 (11.7%)	127 (10.3%)	47 (18.7%)	
No	1317 (88.3%)	1112 (89.8%)	205 (81.4%)	
**Living area**, n (%)				0.142
Rural	820 (55.0%)	668 (53.9%)	152 (60.3%)	
Township	117 (7.9%)	102 (8.2%)	15 (86.0%)	
City	554 (37.2%)	469 (37.9%)	85 (33.7%)	
**Educational attainment**, n (%)				<0.001
No university degree	1176 (78.9%)	948 (76.5%)	228 (90.5%)	
University degree	315 (21.1%)	291 (23.5%)	24 (9.5%)	
**Monthly household income** (Chinese Yuan, CNY), n (%)				<0.001
≤2000	406 (27.2%)	313 (25.3%)	93 (36.9%)	
2001–5000	672 (45.1%)	571 (46.1%)	101 (40.1%)	
5001–10,000	258 (17.3%)	235 (19.0%)	23 (9.1%)	
>10,000	62 (4.2%)	51 (4.1%)	11 (4.4%)	
**Marital status**, n (%)				0.106
Unmarried	67 (4.5%)	62 (5.0%)	5 (2.0%)	
Married (with spouse)	1381 (92.6%)	1141 (92.1%)	240 (95.2%)	
Divorced or widowed	43 (2.9%)	36 (2.9%)	7 (2.8%)	
**Vaccine belief**, n (%)				
Worry about infecting SARS-CoV-2	1092 (73.2%)	901 (72.7%)	191 (75.8%)	0.315
**I think getting the COVID-19 vaccine is important for my health**, n (%)				<0.001
Agree	1319 (88.5%)	1115 (90.0%)	204 (81.0%)	
Neutral	148 (9.9%)	104 (8.4%)	44 (17.5%)	
Disagree	24 (1.6%)	20 (1.6%)	4 (1.6%)	
**I think the COVID-19 vaccine is effective**, n (%)				0.025
Agree	1342 (90.0%)	1127 (91.0%)	215 (85.3%)	
Neutral	133 (8.9%)	100 (8.1%)	33 (13.1%)	
Disagree	16 (1.1%)	12 (1.0%)	4 (1.6%)	
**I think getting the COVID-19 vaccine is important for the health of those around me**, n (%)				0.004
Agree	1366 (91.6%)	1146 (92.5%)	220 (87.3%)	
Neutral	111 (7.4%)	80 (6.5%)	31 (12.3%)	
Disagree	14 (0.9%)	13 (1.1%)	1 (0.4%)	
**I think the COVID-19 vaccine provided by the government is helpful in epidemic prevention**, n (%)				0.382
Agree	1390 (93.2%)	1158 (93.5%)	232 (92.1%)	
Neutral	89 (6.0%)	70 (5.7%)	19 (7.5%)	
Disagree	12 (0.8%)	11 (0.9%)	1 (0.4%)	
**I think COVID-19 vaccination has adverse effects on CLD and treatment**, n (%)				<0.001
Agree	548 (36.8%)	446 (36.0%)	102 (40.5%)	
Neutral	397 (26.6%)	297 (24.0%)	100 (39.7%)	
Disagree	546 (36.6%)	496 (40.0%)	50 (19.8%)	
**I think the COVID-19 vaccine is safe**, n (%)				0.000
Agree	1283 (86.1%)	1086 (87.7%)	197 (78.2%)	
Neutral	191 (12.8%)	139 (11.2%)	52 (20.6%)	
Disagree	17 (1.1%)	14 (1.1%)	3 (1.2%)	

IQR = Q3 − Q1. Autoimmune liver diseases include autoimmune hepatitis, primary biliary cholangitis and primary sclerosing cholangitis. Other liver diseases include liver cancer, liver transplantation, liver damage, drug-induced liver disease, jaundice, Budd–Chiari syndrome and hepatic encephalopathy. Antiviral drugs include entecavir, tenofovir disoproxil fumarate, tenofovir alafenamide fumarate, elbavir graprevir, ledipasvir sofosbuvir, sofosbuvir velpatasvir, tenofovir and adefovir dipivoxil. Immunosuppressants include azathioprine, tacrolimus, cyclosporine, sirolimus and prednisone. Chinese patent medicines include Ruangan Huajian granule, Fuzheng Huayu capsule, AnLuoHuaXianWan and DaHuangZheChongWan. Abbreviations: COVID-19: coronavirus disease 2019; ALD: alcohol-related liver disease, NAFLD: nonalcoholic fatty liver disease.

**Table 2:** Factors associated with unvaccinated status for SARS-CoV-2 vaccines.

Factors	Univariate		Multivariate	
	OR (95%CI)	*p*	OR (95%CI)	*p*
Age ≥ 60 (vs. <60)	3.597 (2.585–5.005)	<0.001	2.731 (1.811–4.12)	<0.001
**Types of CLD**				
Hepatitis B/C (vs. not)	0.424 (0.316–0.57)	<0.001	0.833 (0.537–1.292)	0.415
Cirrhosis (vs. not)	5.977 (4.466–8.00)	<0.001	2.922 (1.972–4.331)	<0.001
Autoimmune liver diseases (vs. not)	2.876 (1.437–5.757)	0.003	1.172 (0.453–3.034)	0.744
**Liver disease course** (vs. <1 year)				
1–5 years	3.558 (2.021–6.262)	<0.001	3.37 (1.782–6.374)	<0.001
5–10 years	3.23 (1.8–5.796)	<0.001	3.236 (1.666–6.286)	0.001
>10 years	2.718 (1.566–4.719)	<0.001	2.792 (1.495–5.216)	0.001
**Treatments (vs. not)**	4.485 (2.523–7.975)	<0.001	2.471 (1.318–4.632)	0.005
Immunosuppressants	5.029 (1.748–14.465)	<0.001	3.764 (1.083–13.08)	0.037
Chinese patent medicine	2.744 (1.998–3.769)	<0.001	1.16 (0.777–1.733)	0.467
**Comorbidities (vs. not)**	3.062 (2.244–4.178)	<0.001	2.004 (1.058–3.798)	0.033
Cardiovascular and cerebrovascular diseases	2.366 (1.58–3.543)	<0.001	0.748 (0.379–1.475)	0.401
Respiratory diseases	3.333 (1.176–9.45)	0.024	0.491 (0.125–1.932)	0.309
Diabetes	3.882 (2.474–6.092)	<0.001	1.101 (0.553–2.192)	0.785
Chronic kidney disease	2.921 (1.139–7.495)	0.026	0.963 (0.315–2.95)	0.948
**Allergy history (vs. not)**	2.007 (1.392–2.896)	<0.001	1.732 (1.107–2.71)	0.016
**No university degree (vs. have)**	2.916 (1.877–4.531)	<0.001	1.959 (1.187–3.231)	0.008
**Not knowledgeable about COVID-19 vaccine considerations (vs. knowledgeable)**	3.158 (2.303–4.332)	<0.001	3.145 (2.253–4.391)	0.001
**Thinking COVID-19 vaccination affects treatment (vs. not and neutral)**	3.54 (2.522–4.968)	<0.001	1.89 (1.284–2.783)	<0.001
**Not receiving doctors’ advice on vaccination (vs. receiving)**	4.508 (3.398–5.981)	<0.001	3.145 (2.253–4.391)	<0.001

Autoimmune liver diseases include autoimmune hepatitis, primary biliary cholangitis and primary sclerosing cholangitis. Immunosuppressants include azathioprine, tacrolimus, cyclosporine, sirolimus and prednisone. Chinese patent medicines include Ruangan Huajian granule, Fuzheng Huayu capsule, AnLuoHuaXian pills and DaHuangZheChong pills.

**Table 3:** Attitudes to the fourth dose of the SARS-CoV-2 vaccine.

Vaccine Type	Number of Participants Who Completed the Third Dose of the Vaccine	Number of Participants Who Accepted the Vaccine	Number of Participants Who Refused the Vaccine	*p*-Value
**Inactivated vaccines, n (%)**	668 (59.9%)	529 (58.3%)	139 (58.9%)	0.409
**Recombinant protein vaccines, n (%)**	465 (39.2%)	372 (41.0%)	93 (39.4%)	
**Mixed vaccines, n (%)**	11 (0.9%)	7 (0.8%)	4 (1.7%)	

## Data Availability

The data will be available upon request.

## Abbreviations

ALD: alcohol-related liver disease; ALT: alanine aminotransferase (ALT); CI: confidence intervals; CLD: chronic liver disease; COVID-19: coronavirus disease 2019; IQR: interquartile range; NAFLD: nonalcoholic fatty liver disease; OR: odds ratios; SARS-CoV-2: severe acute respiratory syndrome coronavirus 2; TBil: total bilirubin.
